# Finding Liebig’s law of the minimum

**DOI:** 10.1002/eap.2458

**Published:** 2021-10-20

**Authors:** Jinyun Tang, William J. Riley

**Affiliations:** ^1^ Earth and Environmental Sciences Area Lawrence Berkeley National Laboratory Berkeley California 94720 USA

**Keywords:** additive model, biogeochemical modeling, biological growth, complementary substrates, law of mass action, law of the minimum, synthesizing unit

## Abstract

Liebig’s law of the minimum (LLM) is often used to interpret empirical biological growth data and model multiple substrates co‐limited growth. However, its mechanistic foundation is rarely discussed, even though its validity has been questioned since its introduction in the 1820s. Here we first show that LLM is a crude approximation of the law of mass action, the state of art theory of biochemical reactions, and the LLM model is less accurate than two other approximations of the law of mass action: the synthesizing unit model and the additive model. We corroborate this conclusion using empirical data sets of algae and plants grown under two co‐limiting substrates. Based on our analysis, we show that when growth is modeled directly as a function of substrate uptake, the LLM model improperly restricts the organism to be of fixed elemental stoichiometry, making it incapable of consistently resolving biological adaptation, ecological evolution, and community assembly. When growth is modeled as a function of the cellular nutrient quota, the LLM model may obtain good results at the risk of incorrect model parameters as compared to those inferred from the more accurate synthesizing unit model. However, biogeochemical models that implement these three formulations are needed to evaluate which formulation is acceptably accurate and their impacts on predicted long‐term ecosystem dynamics. In particular, studies are needed that explore the extent to which parameter calibration can rescue model performance when the mechanistic representation of a biogeochemical process is known to be deficient.

## Introduction

The law of the minimum was proposed by Carl Sprengel as early as 1826 to guide fertilizer use in agricultural practices (Sprengel 1826), and was made popular by Liebig (1840) and later followers as a general rule to interpret biological growth data in various contexts (see van der Ploeg et al. [1999] for an excellent review of the history and development of LLM). LLM states that the growth of an organism is constrained by the most limiting nutrient at that moment. More recently, to address pressing social‐environmental challenges, such as carbon–climate feedbacks and food security, many ecosystem models have adopted LLM to simulate the growth of plants and microorganisms that affect crop yield (under various levels of fertilization), the global carbon cycle (Achat et al. 2016, Lawrence et al. 2019, Zhu et al. 2019), aquatic and ocean biogeochemistry (Degroot 1983, Yool et al. 2011), etc. Notably, LLM is also used to formulate photosynthesis in the commonly applied Farquhar model and its progenitors (e.g., Farquhar et al. 1980, Leuning 1990).

Although the LLM model enjoys widespread popularity, it has been criticized for being deficient in modeling natural and crop plant growth (e.g., Sinclair and Park 1993, Kobe 1996). Further, O’Neill et al. (1989) suggested that they preferred the additive model for its overall better predictions across eleven sets of growth data they analyzed. Later, Kooijman (1998) showed that the synthesizing unit (SU) model can successfully explain the growth pattern measured by Droop (1974), who in his experiment grew algae under two co‐limiting substrates (phosphorus and vitamin B_12_). Moreover, Droop (1974) inferred that the LLM model interpreted his algal growth data better than the multiplicative model (Droop 1973), another formulation (of which the dual Monod kinetics is an example) often used to model biological growth (e.g., Megee et al. 1972, Zinn et al. 2004). However, we recently showed that dual Monod kinetics and single‐substrate Monod kinetics adopt opposite assumptions for the characteristics of the kinetic parameters, rendering the multiplicative Monod kinetics mathematically incapable of consistent upscaling from a single substrate reaction to many‐substrate reactions (Tang and Riley 2017). Nonetheless, to our knowledge, the mechanistic foundation of the LLM model has not been described in the literature, nor has its relationship with other growth models (e.g., the SU model and the additive model) been analyzed.

For both unicellular and multicellular organisms, growth emerges from the interaction between a great number of chemical reactions, most of which are enzyme catalyzed. Meanwhile, it was established more than a century ago that the law of mass action developed for abiotic chemical reactions can also be used to model enzyme reactions (Henri 1903, Michaelis and Menten 1913). Therefore, if we accept the LLM model as a mechanistic representation of biological growth, it should be consistent with the law of mass action. Evaluating this hypothesis should shed light on the limitations of the LLM model in biogeochemical modeling.

In the following, we conduct our analysis under the guidance of following questions: (1) What is the mechanistic foundation of the LLM model? (2) What are the relationships of the LLM model to the SU and additive growth models? And (3) what are the limitations of the LLM model in biogeochemical modeling?

## Methods

### Mechanistic representation of multi‐substrate co‐limited growth

**Fig. 1:**
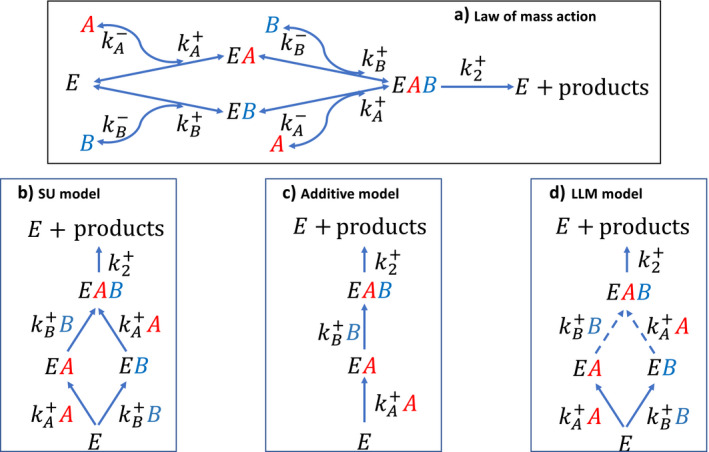
(a) Schematic of *E* working on two complementary substrates *A* and *B* to deliver products, e.g., biomass, gases, or water. (b) Schematic for the synthesizing unit (SU) model. (c) Schematic for the additive model, and we note that it ignores the binding order effect on *EAB* from the binding of *A* and *B* to *E*. (d) Schematic for the Liebig’s law of the minimum (LLM) model, where dashed lines mean the associated substrate is unlimited. The SU model, additive model, and LLM model are approximations to the law of mass action. Forward reaction parameters are designated with superscript “+”, while backward reaction parameters are designated with superscript “−”. The *k* terms represent rate constants for each reaction in appropriate units.

Our analysis below is based on the presumption that the law of mass action is applicable to modeling multiple substrates co‐limited growth. The legitimacy of the law of mass action for simple enzyme systems is well established, for example, by its use to derive various enzyme kinetics under different assumptions (Michaelis and Menten 1913, Cornish‐Bowden 2012, Tang and Riley 2013, 2017). Its applicability for organisms is also phenomenologically supported (e.g., the establishment of Monod kinetics [Monod 1949]). Further, flux balance models based on the law of mass action for the dominant chemical reactions have been shown to successfully represent microbial growth under the assumption of steady‐state proteomic distribution (e.g., Orth et al. 2010, Labhsetwar et al. 2014). Therefore, we hypothesize that its scaling capability enables the law of mass action to model growth at the organism level. Indeed, the law of mass action is widely used in macroecology to model biological growth based on predation and substrate uptake (e.g., Hannon and Ruth 1997). Based on this hypothesis, we assume that biological growth can be conceptually depicted as a central enzyme system (i.e., synthesizing unit as called in Kooijman (1998)) that builds biomass from two incoming complementary substrates *A* and *B*, as formulated in the schema of Fig. 1a.

Applying the law of mass action to the schema in Fig. 1a, and taking mass conservation and the steady‐state approximation for *EA*, *EB*, and *EAB* (i.e., their temporal changes within the time step of biomass production is negligible), we obtain

(1) $$k_{A}^{+}\left[E\right]\left[A\right]+k_{B}^{‐}\left[\mathrm{EAB}\right]=\left(k_{A}^{‐}+k_{B}^{+}\left[B\right]\right)\left[\mathrm{EA}\right]$$

(2) $$k_{B}^{+}\left[E\right]\left[B\right]+k_{A}^{‐}\left[\mathrm{EAB}\right]=\left(k_{B}^{‐}+k_{A}^{+}\left[A\right]\right)\left[\mathrm{EB}\right]$$

(3) $$k_{A}^{+}\left[\mathrm{EB}\right]\left[A\right]+k_{B}^{+}\left[\mathrm{EA}\right]\left[B\right]=\left(k_{A}^{‐}+k_{B}^{‐}+k_{2}^{+}\right)\left[\mathrm{EAB}\right]$$

(4) $${\left[E\right]}_{T}=\left[E\right]+\left[\mathrm{EA}\right]+\left[\mathrm{EB}\right]+\left[\mathrm{EAB}\right]$$

(5) $${\left[A\right]}_{T}=\left[A\right]+\left[\mathrm{EA}\right]+\left[\mathrm{EAB}\right]$$

(6) $${\left[B\right]}_{T}=\left[B\right]+\left[\mathrm{EB}\right]+\left[\mathrm{EAB}\right]$$

where forward reaction parameters are designated with superscript “+” and reverse reaction parameters are designated with superscript “−.” We note that, when applied to biological growth, the kinetic parameters $k_{A}^{+}$ and $k_{B}^{+}$ involve contributions from both physical transport (e.g., diffusion) and other enzymatic processes preparing the substrates *A* and *B* (Berg and Purcell 1977, Tang and Riley 2019). In addition, following previous studies (Kooijman 1998, Brandt et al. 2004), we assume that the forward and reverse reaction parameters for *A* and *B* are independent from the complex status of the central enzyme, e.g., $k_{A}^{+}$ is the same for association between *A* and *E* or between *A* and *EB*. In the following, by taking different approximations in Eqs. (1), (2), (3), (4), (5), (6), we will derive the SU model, the additive model, and the LLM model.

By defining $f_{A}=k_{A}^{+}\left[A\right]$ and $f_{B}=k_{B}^{+}\left[B\right]$, and assuming that $f_{A}⪢k_{B}^{‐}$, $f_{B}⪢k_{A}^{‐}$, and $k_{2}^{+}⪢k_{A}^{‐}+k_{B}^{‐}$ (and also see schema b in Fig. 1), from Eqs. (1), (2), (3), (4), we obtain the SU model (also see Appendix S1)

(7) $$F_{\text{SU}}=\frac{{\left[E\right]}_{T}}{\frac{1}{k_{2}^{+}}+\frac{1}{f_{A}}+\frac{1}{f_{B}}‐\frac{1}{f_{A}+f_{B}}}=\frac{k_{2}^{+}{\left[E\right]}_{T}}{1+\frac{K_{A}}{\left[A\right]}+\frac{K_{B}}{\left[B\right]}‐\frac{1}{\left[A\right]/K_{A}+\left[B\right]/K_{B}}}$$

where $K_{A}=k_{2}^{+}/k_{A}^{+}$, and $K_{B}=k_{2}^{+}/k_{B}^{+}$. Further, we note that by substituting Eqs. 5 and 6 into Eq. 7 and taking a first‐order approximation (with respect to the enzyme‐substrate complexes), one can obtain the SUPECA kinetics (which is generally more accurate than SU kinetics) derived in Tang and Riley (2017). However, because the improved accuracy of SUPECA is significant only when the problem of interest involves interactions with adsorption surfaces (e.g., soil minerals), we will not consider it here.

Next, if we ignore the last term in the denominator of Eq. 7 (or apply the steady state approximation to *EA*, *EB*, and *EAB* according to schema c in Fig. 1, where *EAB* is formed from a serial binding of *A* and *B* to *E*; see Appendix S2), we can derive the additive model

(8) $$F_{\text{ADD}}=\frac{k_{2}^{+}{\left[E\right]}_{T}}{1+\frac{K_{A}}{\left[A\right]}+\frac{K_{B}}{\left[B\right]}}.$$

We note that there is an alternative form to the schema in Fig. 1c, i.e., *EAB* is formed by first binding *E* to *B* and then to *A*. If schema Fig. 1c and its alternative are considered together (when formulating the substrate‐enzyme relationships), we then obtain Fig. 1b, the SU model. We thus find that $‐1/\left(\left[A\right]/K_{A}+\left[B\right]/K_{B}\right)$ in Eq. 7 accounts for the enhanced reaction rate of *F*_SU_ over *F*_ADD_ due to two equivalent reaction pathways.

Last, we formulate the LLM model by taking another approximation to Eq. 8

(9) $$F_{\text{LLM}}=k_{2}^{+}{\left[E\right]}_{T}\cdot \min\left(\frac{\left[A\right]}{K_{A}+\left[A\right]},\frac{\left[B\right]}{K_{B}+\left[B\right]}\right)=k_{2}^{+}{\left[E\right]}_{T}\min\left(\frac{1}{1+K_{A}/\left[A\right]},\frac{1}{1+K_{B}/\left[B\right]}\right).$$

Mathematically, the LLM model is derived by first assuming that substrate *B* is unlimited, which leads to the first term $\left(k_{2}^{+}{\left[E\right]}_{T}/\left(1+K_{A}/\left[A\right]\right)\right)$. Alternatively, assuming that *A* is unlimited leads to the second term $\left(k_{2}^{+}{\left[E\right]}_{T}/\left(1+K_{B}/\left[B\right]\right)\right)$. Finally, the actual growth is taken as the minimum of the two. Conceptually, this derivation can also be understood by applying the steady state approximation to *EA*, *EB*, and *EAB* according to schema d in Fig. 1 (i.e., take the minimum of the rates calculated by the two pathways).

**Fig. 2:**
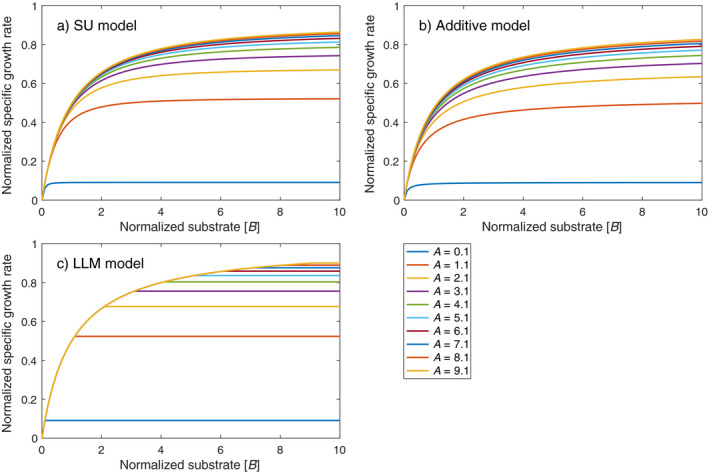
Specific growth rate (normalized by $k_{2}^{+}{\left[E\right]}_{T}$) as a function of substrate concentration *B* (normalized by *K_B_*) for 10 different values of substrate *A* (normalized by *K_A_*).

To provide a visual appreciation of the differences among the SU, additive, and LLM models for two‐substrate co‐limited growth, we compared their functional response curves (of normalized growth rate as a function of the normalized availability of substrate *B* while keeping substrate *A* normalized at nine different levels; Fig. 2). There we see that the SU and additive models are quite similar (which we will see again when these models are evaluated using observed growth data) in a way that resembles the classic Michaelis‐Menten kinetics, whereas the LLM model is quite different in that it has a maximum when the growth is limited by the controlling parameter (i.e. availability of substrate *A* in Fig. 2).

For growth that is co‐limited by more than two complimentary substrates, the SU model can be extended by applying the renewal theory (Kooijman 1998)

(10) $$\begin{array}{ll} & F_{\text{SU}}=k_{2}^{+}{\left[E\right]}_{T}\\ & {\left(\begin{array}{c} 1+\sum_{j}\frac{k_{2}^{+}}{f_{j}}‐\sum_{j_{2}>j_{1}=1}\frac{k_{2}^{+}}{f_{j_{1}}+f_{j_{2}}}+\cdots \\ +{\left(‐1\right)}^{m‐1}\sum_{j_{m}>\cdots >j_{2}>j_{1}=1}\frac{k_{2}^{+}}{f_{j_{1}}+f_{j_{2}}+\cdots +f_{j_{m}}}+\cdots \end{array}\right)}^{‐1} \end{array}$$

where $f_{j}=k_{\mathrm{Sj}}^{+}\left[S_{j}\right]$.

The corresponding additive model is

(11) $$F_{\text{ADD}}=\frac{k_{2}^{+}{\left[E\right]}_{T}}{1+\sum_{j}k_{2}^{+}/f_{j}}=\frac{k_{2}^{+}{\left[E\right]}_{T}}{1+\sum_{j}K_{j}/S_{j}}$$

with $K_{j}=k_{2}^{+}/k_{j}^{+}$ for substrate *S_j_*, and the corresponding LLM model is

(12) $$F_{\text{LLM}}=k_{2}^{+}{\left[E\right]}_{T}\cdot \min_{j}\left(\frac{1}{1+K_{j}/S_{j}}\right).$$

We note that these three models with more than two complimentary substrates can also be understood (and derived) using similar schemas as presented in Fig. 1.

From the above derivations, we note that the two‐substrate SU model ignores the dissociation terms in the law of mass action equations (i.e., terms related to $k_{A}^{‐}$ and $k_{B}^{‐}$ in Eqs. (1), (2), (3)), while the two‐substrate additive model ignores the parallel interaction terms of the SU model (i.e., $‐1/\left(\left[A\right]/K_{A}+\left[B\right]/K_{B}\right)$ in Eq. 7), and the two‐substrate LLM model calculates the reaction rate using the minimum of the asymptotes along the two substrate axes of the additive model. We thus hypothesize that, with identical parameter values, the SU model, the additive model, and the LLM model will approximate the law of mass action with decreasing accuracy. Below we show that even though all three models are approximations to the law of mass action, the more dramatic loss of approximation accuracy in the LLM model (when compared to the other two models) implies that parameter calibration cannot always make up its structural deficiency in modeling biological growth.

### Empirical data for evaluating the three models

Since the models derived above are intended to be applied to uni‐ or multicellular organisms, we evaluate them against two types of observed responses, one for unicellular microbes (i.e., algae) and one for plants. In total, we identified eight sets of data from two publications to evaluate the capability of these three models in predicting two‐substrate co‐limited growth. The first two data sets are from Droop (1974), who conducted batch experiments by growing algae (*Monochrysis*) on different supply levels of phosphorus and vitamin B_12_. The experiments characterized the growth of separate populations of slow‐ and fast‐adapted cells. In the data set, nutrient availability was measured as cellular quota (i.e., nutrient concentration in the cell), and growth was measured as dilution rate (calculated as the ratio between the media flow rate into the experimental container and the culture volume). The other data sets are from Shaver and Melillo (1984), who conducted pot experiments by growing *Carex lacustris*, *Calamagrostis canadensis*, and *Typha latifolia* with nitrogen and phosphorus fertilizers. The Shaver and Mellilo data are presented as measured biomass harvested at two times (i.e., 5 and 7 months after planting, indicated by H1‐ and H2‐, respectively), with their corresponding nitrogen and phosphorus additions applied factorially.

We inferred the best posterior parameters by fitting these three models (in their two‐substrate forms) to the above data sets using the *fminsearch* function from MATLAB (R2020b) to minimize the difference between predicted and measured growth data. For each model, the goodness of fit is reported as root mean square error (RMSE), a linear regression of measured vs. predicted growth rate, and *R* ^2^ value. Finally, we note that for algae, parameter $k_{2}^{+}$ designates the actual specific growth rate (d^−1^), while for plants, $k_{2}^{+}$ represents $k_{2}^{+}{\left[E\right]}_{T}$ integrated over their respective growth periods until harvest.

## Results

**Fig. 3:**
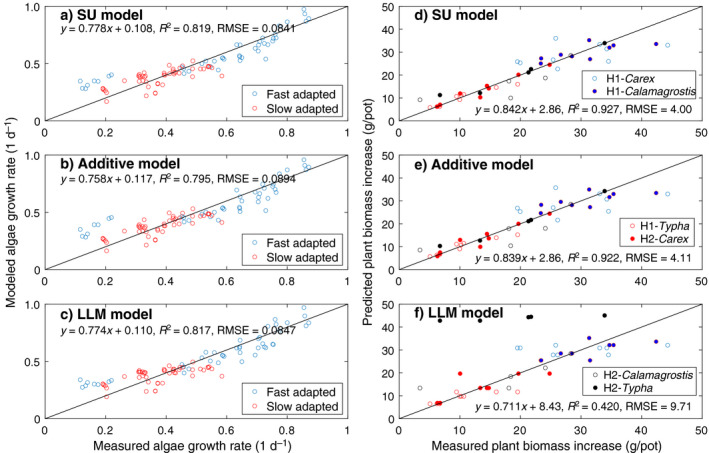
Model predicted and measured growth rates for (a–c) the Droop algae data and (d–f) the Shaver and Mellilo plant data. The Droop data have fast‐ and slow‐adapted algae groups. The Shaver and Melillo data have six groups of measurements, with H1‐ meaning data from the first harvest, and H2‐ meaning data from the second harvest. For each panel, the linear regression was done for all data involved. Additionally, we note that the legends in panels d, e, and f should be read together.

Using the inferred parameters for the three models (Table 1), we found all three models fit the observed algal growth almost equally well (left panels in Fig. 3), with their performances (based on RMSE and the regression slope) from best to worst ranked as the SU model, the LLM model, and the additive model. In contrast, for the data set of plant growth, the SU model performed slightly better than the additive model, while the LLM model fit poorly, particularly for the growth data of *Typha* (identified as H2‐*Typha* in Fig. 3f) collected in the second harvest.

**Table 1:** Inferred parameter values for the algal and plant data sets.

Model	ID	$k_{2}^{+}$	*K_A_*	*K_B_*
SU model				
Algae: fast adapted	1	4.15	4.72	27.8
Algae: slow adapted	2	0.842	0.623	4.20
Plant: H1‐*Carex*	3	38.9	1.11	0.148
Plant: H2‐*Carex*	4	37.1	0.624	0.117
Plant: H1‐*Calamagrostis*	5	20.4	5.05	0.280
Plant: H2‐*Calamagrostis*	6	38.5	9.94	0.697
Plant: H1‐*Typha*	7	38.8	2.59	0.940
Plant: H2‐*Typha*	8	45.4	2.40	0.890
Additive model				
Algae: fast adapted	1	2.49 (0.600)	2.09 (0.443)	11.7 (0.421)
Algae: slow adapted	2	0.811 (0.963)†	0.475 (0.762)†	3.27 (0.779)†
Plant: H1‐*Carex*	3	38.9 (1.00)†	0.902 (0.813)†	0.129 (0.872)†
Plant: H2‐*Carex*	4	36.9 (0.995)†	0.481 (0.771)†	0.0920 (0.786)†
Plant: H1‐*Calamagrostis*	5	19.2 (0.941)	4.10 (0.812)	0.170 (0.607)
Plant: H2‐*Calamagrostis*	6	40.4 (1.05)†	9.38 (0.944)†	0.539 (0.773)†
Plant: H1‐*Typha*	7	40.3 (1.03)†	2.10 (0.811)†	0.904 (0.962)†
Plant: H2‐*Typha*	8	48.3 (1.06)†	2.19 (0.91)†	0.881 (0.990)†
LLM model				
Algae: fast adapted	1	17.2 (4.14)	24.6 (5.21)	154 (5.54)
Algae: slow adapted	2	0.748 (0.888)†	0.536 (0.860)†	3.76 (0.895)†
Plant: H1‐*Carex*	3	32.7 (0.841)	0.129 (0.116)	0.0571 (0.386)
Plant: H2‐*Carex*	4	37.0 (0.997)†	0.663 (1.06)†	0.148 (1.27)†
Plant: H1‐*Calamagrostis*	5	13.0 (0.637)	2.21 (0.438)	0.0266 (0.0950)
Plant: H2‐*Calamagrostis*	6	25.7 (0.667)	6.12 (0.616)	0.00965 (0.0138)
Plant: H1‐*Typha*	7	33.1 (0.853)	3.27 (1.26)	0.261 (0.278)
Plant: H2‐*Typha*	8	45.4 (1.00)	0.0529 (0.0220)	0.0196 (0.0220)

Notes

For algal growth data from Droop (1974), the units of $k_{2}^{+}$, *K_A_* (phosphorus), and *K_A_* (vitamin B_12_) are d^−1^, nmol·(L P)^−1^·(million cells)^−1^, and fmol·(L B_12_)^−1^·(million cells)^−1^, respectively. For plant data from Shaver and Melillo (1984), the units of $k_{2}^{+}$ (which integrates the contribution of [*E*]*_T_* in the three models), *K_A_* (nitrogen), and *K_B_* (phosphorus) are g dry biomass/pot, g N/pot, and g P/pot, respectively. The numbers in parentheses for the additive model and the Liebig’s law of the minimum (LLM) model measure the relative magnitude of a parameter with respect to its corresponding value for the synthesizing unit (SU) model.

† Entries within 20% of the corresponding parameters inferred for the SU model.

**Fig. 4:**
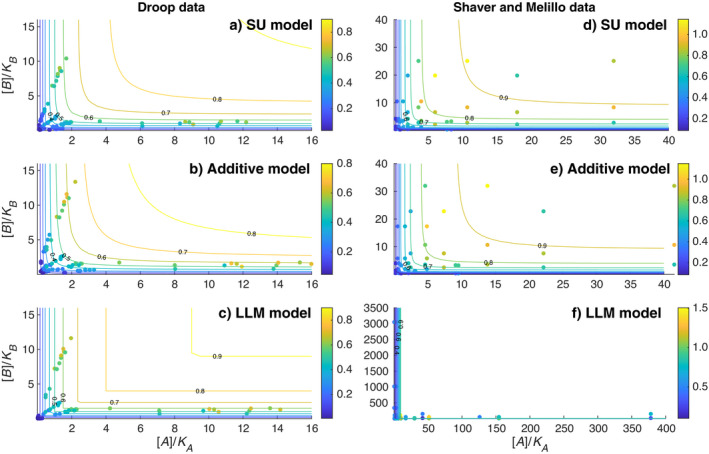
Observed growth rates in the normalized contour plots of different models: (a–c) for algal growth data; (d–f) for plant growth data. In each plot, the observed growth rates are normalized with the maximum growth rates inferred from the model and each substrate is normalized with its corresponding affinity parameter. The contour lines are derived from the relevant Eqs. (7), (8), (9) for each model. The color bars are the relative growth rates. In panel f, the poor performance of the LLM model forces us to use different axes limits from those in panels d and e. An expanded version for better visual of data points around the origins is available in Appendix S3: Fig. S1.

As can be inferred from Eqs. (7), (8), (9), when viewed as functions of the normalized substrate concentrations [*A*]/*K_A_* and [*B*]/*K_B_*, the normalized growth rates $F_{\text{SU}}/k_{2}^{+}$, $F_{\text{ADD}}/k_{2}^{+}$, and $F_{\text{LLM}}/k_{2}^{+}$ are independent from the characteristics of the experimental organisms. Therefore, for each model, all algal and plant growth data can be considered to follow the same contour of the normalized growth rates (and similarly for plant growth data). This representation enables an alternative view of the growth data in the context of different models. We thus normalized the measured growth rates with their correspondingly inferred maximum growth rate (Table 1), and then plotted them together with the contours of the normalized growth rate predicted by each model as a function of the normalized substrate availability (Fig. 4). The results show that the algal growth data mostly exist near the origin or are close to the two axes (Fig. 3a, b, c), while the plant growth data spread out more (Fig. 4d, e). Overall, we find that the LLM model worked well for the Droop data (which calculated algal growth rates based on cellular nutrient quota), but not for the Shaver and Melillo data (which calculated plant growth rates based on nutrient uptake fluxes). In particular, the LLM model almost failed completely for the plant growth data (Fig. 4f; see also Appendix S3: Fig. S1). This result suggests that when evaluated as a function of external substrate supply, the plants were experiencing nitrogen and phosphorus co‐limitation in the experiments by Shaver and Melillo. In contrast, likely because of the small size of unicellular algae, the limited variability of nutrient quota in the Droop data is not able to differentiate among the three growth models.

**Fig. 5:**
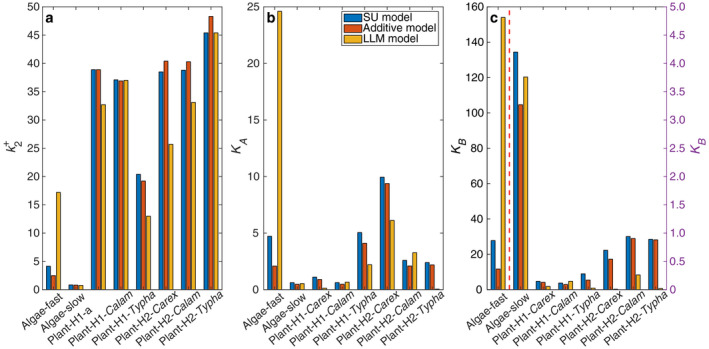
Comparison of inferred parameters of the three models for the eight data sets: panel a is for growth rate, panels b and c are for substrate affinity. The parameter values are in Table 1. For panel c, except for fast‐adapted algae (separated by the red dashed line), parameter values follow the right *y*‐axis. *Calam* is *Calamagrostis* abbreviated.

All three inferred parameters (Table 1; Fig. 5) have similar magnitudes between the SU model and the additive model (as expected based on the similar functional response curves in Fig. 2 and contours in Fig. 3). However, the LLM model inferred quite different parameters. When measured in terms of relative difference as compared to corresponding parameters of the SU model (which is supposed to be the most accurate among the three models), six out of eight sets of the parameters are within 20% relative difference for the additive model. In contrast, only two sets of the parameters are within 20% relative difference for the LLM model. For the fast‐adapted algal group (Table 1 and also see Fig. 5), the additive model inferred parameters are about half those of the SU model, because the interaction term ($1/\left(\left[A\right]/K_{A}+\left[B\right]/K_{B}\right)$) of the SU model is of comparable magnitude to other terms. In contrast, the LLM model inferred parameters are more than four times as large as those of the SU model. For plants, the maximum growth rates ($k_{2}^{+}$) are mostly of the same magnitude among the three models, whereas the nitrogen affinity parameters (*K_A_*) for the LLM model are much smaller than the other two models for H1‐*Carex* and H2‐*Typha*, while the phosphorus affinity parameters (*K_B_*) for the LLM model are much smaller than for the other two models for H1‐*Calamagrostis*, H2‐*Calamagrostis*, and H2‐*Typha*.

## Discussion

In the following, we address the three main questions that motivated this analysis.

### 1 What is the mechanistic foundation of the LLM model?

We found that the LLM model can be derived from the first principle‐based law of mass action by imposing the condition that all but one substrate is of unlimited supply at any particular time. This simplification makes the LLM model less accurate than the SU model and additive model in handling growth co‐limited by multiple substrates, particularly when the relative supply of the complimentary substrates is comparable (e.g., plants in the right column of Fig. 4).

### 2 What is the relationship of the LLM model to the SU and additive growth models?

**Fig. 6:**
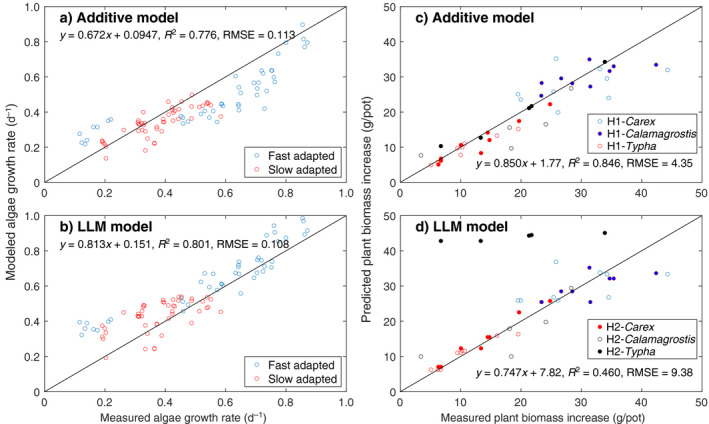
Model predicted with measured growth rate for (a and b) Droop’s algae data and Shaver and (c and d) Mellilo’s plant data. All models used parameters from the corresponding SU model.

In the mathematical derivations above, we showed that the LLM model can be viewed as a crude approximation to the law of mass action model, and thus to the more accurate SU and additive models. Theoretically, if the law of mass action model is the reference growth model (as we have assumed), under a wide range of conditions, the SU model is the most accurate, followed by the additive model and the LLM model. Moreover, the SU and additive models have qualitatively similar responses to changes in the availability of co‐limiting substrates (as can be seen from the similarity between the functional response and contour plots for the SU and additive models; Figs. 2, 4), whereas the LLM model behaves differently under co‐limiting conditions. Importantly, all three models (as shown in Eqs. 7–9) share the same parameters for application. However, because of their different accuracy in approximating the law of mass action model, performance degradation should in general be expected when true model parameters (as assumed to be associated with the most accurate SU model) are used (e.g., Fig. 3 vs. Fig. 6), and occasionally the degradation will even make the LLM model unacceptable (Fig. 6d). Therefore, we assert that although calibration can sometimes make a biogeochemical model that uses the LLM model perform well for a particular benchmark data set, calibration cannot always make up for deficiencies in the model’s structural accuracy, even if the desired processes (i.e., multiple substrates co‐limited growth here) are nominally represented in the model.

### 3 What are the limitations of the LLM model in biogeochemical modeling?

While the LLM model has the same number of parameters as the SU and additive models, when it is used to model growth directly as a function of substrate fluxes (as in the plant example above), the organisms must be of fixed elemental stoichiometry. This restriction can be clearly demonstrated with the León‐Tumpson model (1975)

(13) $$\frac{dN_{i}}{\mathrm{dt}}=N_{i}\left(\min_{j}\left\{\frac{g_{\mathrm{ij}}\left(R_{j}\right)}{q_{\mathrm{ij}}}\right\}‐D_{i}\right),i=1,\cdots ,n,j=1,\cdots ,m$$

(14) $$\frac{dR_{j}}{\mathrm{dt}}=f_{j}\left(R_{j}\right)‐\sum_{i}q_{\mathrm{ij}}\left(\min_{j}\left\{\frac{g_{\mathrm{ij}}\left(R_{j}\right)}{q_{\mathrm{ij}}}\right\}\right)N_{i}$$

where population *N_i_* (as biomass) grows on *m* perfectly complementary substrates *R_j_* (here “perfectly complementary” means no growth when any substrate is missing), which are supplied externally at rates $f_{j}\left(R_{j}\right)$. The potential uptake rate by population *N_i_* of substrate *R_j_* is *g_ij_*, with a corresponding biomass conversion factor *q_ij_* (i.e., one unit of biomass for *N_i_* requires *q_ij_* unit of substrate *j*). *D_i_* is the death rate of population *N_i_*. The model applies LLM to compute the growth rate of population *N_i_* as $\min_{j}\{g_{\mathrm{ij}}\left(R_{j}\right)/q_{\mathrm{ij}}\}$ in Eq. 13, which is then used to update the availability of substrate *R_j_* in Eq. 14.

In the León‐Tumpson model, once the growth rate of population *N_k_* is determined by the most limiting substrate *j_k_*, the uptake of any other substrate *j* (by *N_k_*) is determined by $g_{kj_{k}}$ and the ratio $q_{\mathrm{kj}}/q_{kj_{k}}$, rather than by $q_{\mathrm{kj}}$; i.e., the uptake of substrate *j* is $g_{kj_{k}}q_{\mathrm{kj}}/q_{kj_{k}}$. However, Danger et al. (2008) pointed out that this restriction of fixed elemental stoichiometry of population *N_k_* will potentially result in a situation that while population *N_k_* is limited by substrate *j_k_*, the whole system is limited by another substrate due to other mechanisms (e.g., competition, symbiosis). Alternatively, we infer that during a numerical integration step when the uptake fluxes from all populations are summed for each substrate, there exists a possibility that the whole population is limited by substrate *j*, while a specific population *N_k_* is limited by another substrate *j_k_*, creating a conundrum on the validity of using LLM model for the growth of an individual population. Therefore, they asserted (and as we inferred here) that LLM does not scale from individuals to a community. They corroborated this inference with batch experiments of a bacterial community. In a related study, Gorban et al. (2011) asserted that because biological organisms are generally adaptive, they typically will not be limited by a single substrate for long, (in contrast to the implication in the LLM model) creating “law of the minimum paradoxes.” In summary, these previous studies and our results show that, when growth is computed directly from substrate uptake fluxes, the success of the LLM model to interpret a particular observational data set of growth rates (without considering ecological interaction) through parameter calibration does not justify its use as a good biological growth model in a sophisticated ecological context. Rather, such an application of the LLM model (as in the León‐Tumpson model that involves population dynamics and substrate competition) conflicts with the capability of biological organisms to adapt and evolve under environmental stresses and ecological interactions.

All living organisms consist of a core set of macromolecules that are of different elemental stoichiometry and small size molecules of relatively low concentrations (supporting metabolism while not creating osmotic stress; Lodish et al. [1999]). Therefore, cellular elemental stoichiometry is unlikely to be fixed under fluctuating nutrients availability. Indeed, observations indicate that even single cellular organisms can store nutrients for later use when these nutrients are scarce (aka luxury uptake; e.g., Madigan et al. 2009, Powell et al. 2009). Moreover, since multicellular organisms can be viewed as a community of many unicellular organisms, their uptake and use of substrates are more difficult to synchronize than single cellular organisms, making variable elemental stoichiometry the rule rather than the exception for fungi, plants, and animals (e.g., Elser et al. 2000).

In summary, if LLM is used to model biological growth, it should only be applied to the cellular quota of the complementary nutrients, i.e., the modeled organism must be explicitly represented with nutrient storage pools and flexible stoichiometry. This approach is how Droop (1974) used the LLM model to interpret his algae growth data, and it is similarly adopted by the ecosystem model *ecosys* (Grant 2013) to represent carbon, nitrogen, and phosphorus co‐regulated plant and microbial growth. However, we found (in Fig. 5 and Table 1) that applying LLM to cellular nutrient quota‐based growth may still result in posterior parameters that differ significantly from those inferred with the mechanistically more accurate SU model, even though the LLM model may still fit the observations reasonably well (Fig. 6b). Nevertheless, it will be interesting and valuable to compare the SU, additive, and LLM model implementations within a complex biogeochemical model that represents organisms with flexible stoichiometry feeding on a variety of different substrates, and evaluate how simulated ecosystem structure and biogeochemistry are influenced by their differences. In particular, we ask, can parameter calibration always rescue model performance when the deficiency in mechanistic representation is known to be significant?

## Supporting information

Appendix S1

Appendix S2

Appendix S3
