# Supplementary material for: Finding Liebig’s law of the minimum
Source: Ecol Appl. 2021 Oct 20;31(8):e02458. doi: 10.1002/eap.2458 (PMC9285345; doi:10.1002/eap.2458)
Supplement: Supplementary file 1 — Appendix S1 [file EAP-31-0-s003.pdf]

Supporting Information. Tang, J., and W.J. Riley. 2021. Finding Liebig's law of the minimum. Ecological Applications.

Appendix S1. Derivation of equation (7) based on Figure 1b.

The dynamic equations that follow the schema in Figure 1b are

$$\frac{d[EA]}{dt} = k_A^+[E][A] - k_B^+[B][EA], \quad \text{(S1)}$$

$$\frac{d[EB]}{dt} = k_B^+[E][B] - k_A^+[A][EB], \quad \text{(S2)}$$

$$\frac{d[EAB]}{dt} = k_A^+[EB][A] + k_B^+[EA][B] - k_2^+[EAB], \quad \text{(S3)}$$

$$[E]_T = [E] + [EA] + [EB] + [EAB], \quad \text{(S4)}$$

where subscript $_T$ means total concentration.

By aid of the steady-state approximation, equations (S1)-(S3) can be rewritten as

$$k_B^+[B][EA] = k_A^+[E][A], \quad \text{(S5)}$$

$$k_A^+[A][EB] = k_B^+[E][B], \quad \text{(S6)}$$

$$k_2^+[EAB] = k_A^+[EB][A] + k_B^+[EA][B]. \quad \text{(S7)}$$

By solving $[EA]$ from (S5), $[EB]$ from (S6), and then entering the result into (S7) to solve for $[EAB]$, we obtain

$$[EA] = \frac{k_A^+[A]}{k_B^+[B]}[E], \quad \text{(S8)}$$

$$[EB] = \frac{k_B^+[B]}{k_A^+[A]}[E], \quad \text{(S9)}$$

$$[EAB] = \frac{(k_A^+[A]+k_B^+[B])}{k_2^+}[E]. \quad \text{(S10)}$$

When equations (S8)-(S10) are entered into (S4), we have

$$[E] = \frac{[E]_T}{1+\frac{k_A^+[A]}{k_B^+[B]}+\frac{k_B^+[B]}{k_A^+[A]}+\frac{(k_A^+[A]+k_B^+[B])}{k_2^+}}, \quad \text{(S11)}$$

which when combined with (S10) leads to

$$[EAB] = \frac{[E]_T}{1+\frac{k_2^+}{k_B^+[B]}+\frac{k_2^+}{k_A^+[A]}-\frac{k_2^+}{(k_A^+[A]+k_B^+[B])}}, \quad \text{(S12)}$$

from which equation (7) in the main text can be obtained by calculating $k_2^+[EAB]$.
