# Supplementary material for: Finding Liebig’s law of the minimum
Source: Ecol Appl. 2021 Oct 20;31(8):e02458. doi: 10.1002/eap.2458 (PMC9285345; doi:10.1002/eap.2458)
Supplement: Supplementary file 2 — Appendix S2 [file EAP-31-0-s002.pdf]

Supporting Information. Tang, J., and W.J. Riley. 2021. Finding Liebig's law of the minimum. Ecological Applications.

Appendix S2. Derivation of equation (8) of Figure 1c.

The dynamic equations that follow the schema in Figure 1c are

$$\frac{d[EA]}{dt} = k_A^+[E][A] - k_B^+[B][EA], \quad \text{(S1)}$$

$$\frac{d[EAB]}{dt} = k_B^+[EA][B] - k_2^+[EAB], \quad \text{(S2)}$$

$$[E]_T = [E] + [EA] + [EAB]. \quad \text{(S3)}$$

Now by applying the steady-state approximation to equations (S1) and (S2) and doing some re-arrangement, we have

$$[EA] = \frac{k_A^+[A]}{k_B^+[B]}[E], \quad \text{(S4)}$$

$$[EAB] = \frac{k_B^+[B]}{k_2^+}[EA] = \frac{k_A^+[A]}{k_2^+}[E]. \quad \text{(S5)}$$

When equations (S4) and (S5) are entered into (S3), we find

$$[E] = \frac{[E]_T}{1+\frac{k_A^+[A]}{k_B^+[B]}+\frac{k_A^+[A]}{k_2^+}}, \quad \text{(S6)}$$

which when combined with equation (S5) results in

$$[EAB] = \frac{[E]_T}{1+\frac{k_2^+}{k_A^+[A]}+\frac{k_2^+}{k_B^+[B]}}, \quad \text{(S7)}$$

from which equation (8) in the main text can be obtained by calculating $k_2^+[EAB]$.
