# Supplementary material for: Finding Liebig’s law of the minimum
Source: Ecol Appl. 2021 Oct 20;31(8):e02458. doi: 10.1002/eap.2458 (PMC9285345; doi:10.1002/eap.2458)
Supplement: Supplementary file 3 — Appendix S3 [file EAP-31-0-s001.pdf]

Supporting Information. Tang, J., and W.J. Riley. 2021. Finding Liebig's law of the minimum. Ecological Applications.

Appendix S3. Zoom-in version of Figure 4

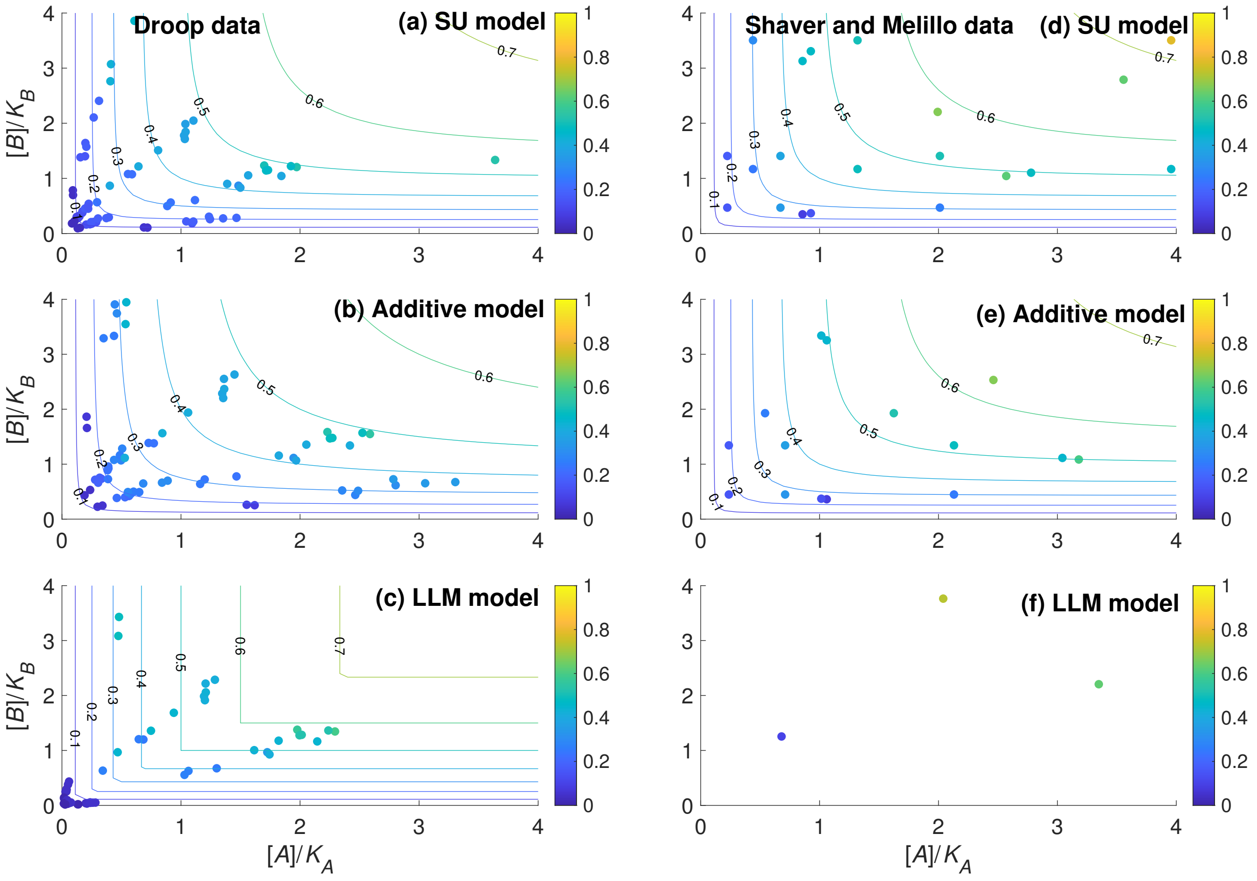

Figure S1. In each plot, the observed growth rates are normalized with the maximum growth rates inferred from the model, and each substrate is normalized with its corresponding affinity parameter. The contour lines are derived from the relevant equations (7, 8, and 9) for each model. The color bars are the relative growth rates. In panel (**f**), the poor performance of the LLM model resulted in no contour lines around the origin.
